# Molecular Genetic Analysis of 103 Sporadic Colorectal Tumours in Czech Patients

**DOI:** 10.1371/journal.pone.0024114

**Published:** 2011-08-25

**Authors:** Peter Vasovcak, Kristyna Pavlikova, Zdenek Sedlacek, Petr Skapa, Martin Kouda, Jiri Hoch, Anna Krepelova

**Affiliations:** 1 Department of Biology and Medical Genetics, Charles University 2nd Faculty of Medicine and University Hospital Motol, Prague, Czech Republic; 2 Department of Pathology and Molecular Medicine, Charles University 2nd Faculty of Medicine and University Hospital Motol, Prague, Czech Republic; 3 Department of Surgery, Charles University 2nd Faculty of Medicine and University Hospital Motol, Prague, Czech Republic; Ohio State University Medical Center, United States of America

## Abstract

The Czech Republic has one of the highest incidences of colorectal cancer (CRC) in Europe. To evaluate whether sporadic CRCs in Czech patients have specific mutational profiles we analysed somatic genetic changes in known CRC genes (*APC*, *KRAS*, *TP53*, *CTNNB1*, *MUTYH* and *BRAF*, loss of heterozygosity (LOH) at the *APC* locus, microsatellite instability (MSI), and methylation of the *MLH1* promoter) in 103 tumours from 102 individuals. The most frequently mutated gene was *APC* (68.9% of tumours), followed by *KRAS* (31.1%), *TP53* (27.2%), *BRAF* (8.7%) and *CTNNB1* (1.9%). Heterozygous germline *MUTYH* mutations in 2 patients were unlikely to contribute to the development of their CRCs. LOH at the *APC* locus was found in 34.3% of tumours, MSI in 24.3% and *MLH1* methylation in 12.7%. Seven tumours (6.9%) were without any changes in the genes tested. The analysis yielded several findings possibly specific for the Czech cohort. Somatic *APC* mutations did not cluster in the mutation cluster region (MCR). Tumours with MSI but no *MLH1* methylation showed earlier onset and more severe mutational profiles compared to MSI tumours with *MLH1* methylation. *TP53* mutations were predominantly located outside the hot spots, and transitions were underrepresented. Our analysis supports the observation that germline *MUTYH* mutations are rare in Czech individuals with sporadic CRCs. Our findings suggest the influence of specific ethnic genetic factors and/or lifestyle and dietary habits typical for the Czech population on the development of these cancers.

## Introduction

Colorectal cancer (CRC) is the second most common form of cancer in Europe, and the Czech Republic has the second highest CRC incidence and mortality among 38 European countries [1]. The reasons for this are unknown and can include both genetic and environmental factors. Hereditary cancer susceptibility syndromes account for no more than 5% of CRC cases [2]. The major autosomal dominant disorders with a high risk of CRC include Lynch syndrome (hereditary non-polyposis colorectal cancer, HNPCC), familial adenomatous polyposis (FAP), Peutz-Jeghers syndrome (PJS) and juvenile polyposis (JP) [3]. *MUTYH* associated polyposis (MAP) is an autosomal recessive hereditary CRC predisposition [4]. The incidence of germline *MUTYH* mutations in Czech FAP negative sporadic CRC patients is lower compared to other European countries [5], [6], and there seems to be no increased incidence of the autosomal dominant forms either.

About 75% CRCs are sporadic, occurring in individuals with no remarkable family history of the disease. Dietary and other lifestyle-related and environmental factors are supposed to play an important role in the aetiology of this form of CRC. Sporadic CRCs have even more biological variables compared to hereditary CRCs. Most sporadic CRCs have mutations in the *APC* gene [7], and an increased rate of G∶C>T∶A transversions in *APC* can also reveal “hidden” MAP patients [4]. Similarly defects in several other pathways result in other specific mutation signatures [8]. *APC* negative tumours can carry *CTNNB1* gene mutations [9], [10]. The mutation status of the *KRAS* and *TP53* genes, two other key players in CRC [11], can reflect carcinogen exposure and reveal the tumour aetiology [12]. Microsatellite instability (MSI) is found in about 15-20% of CRCs; 3-5% are associated with Lynch syndrome and the remaining are sporadic [13]. MSI is associated with *MLH1* promoter methylation, somatic *BRAF* mutations, and has an inverse relationship with *APC* mutations [14], [15]. Thus, the molecular genetic landscape of CRC is rather complex. However, its exploration is a prerequisite for personalized molecular medicine and identification of biomarkers for early detection of tumours, risk stratification, prognosis and prediction of treatment responses [16].

The aim of our study was to contribute to the understanding of CRC tumorigenesis by the analysis of most genes known to be implicated in CRC, and by correlating the molecular genetic profiles of the tumours with their clinical and histopathological data. We also focused on the molecular genetic features of the Czech CRC patients, because we hypothesized that their high incidence and mortality could be accompanied by specific mutation profiles, which could reflect possible specific ethnic, geographical, dietary or lifestyle factors. To this aim we analysed the complete coding region of the *APC* gene and loss of heterozygosity (LOH) at the *APC* locus, the *CTNNB1, MUTYH, KRAS* and *TP53* genes, as well as MSI, methylation status of the *MLH1* promoter, and *BRAF* mutations in CRCs from 102 Czech patients.

## Materials and Methods

### Ethics Statement

The study was based on informed consent and approval of the local ethics committee.

### Patients

The samples were obtained from unselected consecutive patients who had undergone curative surgical resection for primary colorectal cancer at the Department of Surgery, University Hospital Motol, Prague, Czech Republic. Because of our focus on sporadic CRC patients, we excluded individuals with family history of CRC disease and/or presence of polyps. Also excluded were patients who received preoperative radiotherapy, patients with low quality of the DNA sample, and patients in whom no matching mucosa sample was available. Finally 103 tumours and matching normal tissues were collected from 102 CRC patients (51 males and 51 females, age at tumour onset 13-86 years, median 64 years) at the Department of Surgery, University Hospital Motol, Prague, Czech Republic. Fifty-six patients were from Prague and the rest were from all regions of the Czech Republic. All tumours were fresh-frozen at -70°C at colectomy. A minimum of 85% of neoplastic tissue was present in each resected sample as assessed by a pathologist.

### Mutation analysis

DNA was prepared using the Genomic DNA Purification Kit (Gentra Systems, Minneapolis, MI, USA) according to the manufacturer instructions. *APC* exons 1-15, *TP53* exons 2-10, *CTNNB1* exon 3 and *KRAS* exons 1-2 were amplified in PCR reactions containing 20 mM Tris-HCl (pH 8.0), 1 mM DTT, 0.1 mM EDTA, 100 mM KCl, 0.5% (v/v) Nonidet P40, 0.5% (v/v) Tween 20, 50% (v/v) glycerol, 200 µM dNTPs, 1 U of Taq Polymerase (Fermentas, Glen Burnie, MD, USA), 3 pmol of each primer, 1 µl of 10x LCGreen Plus Dye (Idaho Technology, Salt Lake City, UT, USA) and 20 ng of DNA in a total volume of 10 µl for 1 min at 95°C, 45 cycles of 1 min at 95°C, 30 s annealing, 30 s at 72°C, and then 7 min at 72°C. Heteroduplexes were formed by heating the PCR products to 95°C for 2 min and cooling down to 4°C, and subjected to high resolution melting (HRM) analysis using LightScanner (Idaho Technology). *BRAF* exon 15 and *MUTYH* exons 6-8, 12 and 13-14 were sequenced directly from PCR amplicons prepared as above but with 10 pmol of each primer, no dye, in a total volume of 30 µl for 1 min at 95°C, 32 cycles of 1 min at 95°C, 1 min annealing, 1 min at 72°C, and then 7 min at 72°C. Annealing temperatures, MgCl_2_ concentrations and primer sequences are available upon request. PCR products with suspected variations identified by HRM were purified using the SureClean PCR purification kit (Bioline, London, UK) and sequenced in both directions using the BigDye Terminator v3.1 Cycle Sequencing kit on an ABI 3130 Genetic Analyser (Applied Biosystems, Foster City, CA, USA). Somatic mutations found in tumours were also analysed in the corresponding mucosa to assess their germline status. The functional impact of APC mutations was predicted using Polyphen [17]. Bioinformatic analysis also used the NCBI dbSNP database (http://www.ncbi.nlm.nih.gov/snp), the UMD p53 Mutation Database (UMD, http://p53.free.fr/Database/p53_database.html, The p53 Handbook 2.0), and the Leiden Open Variation Database (LOVD, v.2.0 Build 29, http://www.insight-group.org/mutations/). In tumours with 2 or more *APC* mutations separated by a distance of ≤5 kb the phase of the mutations was analysed using allele-specific PCR and sequencing. The primer sequences are available upon request.

### LOH analysis

LOH at the *APC* locus was tested using the microsatellite marker D5S346 and capillary electrophoresis on an ABI 3130 Genetic Analyser. Allelic loss was scored if the area under one allelic peak in the tumour was reduced by 50% or more relative to the other allele, after correcting for the ratio of allelic peak areas in normal DNA. Samples with constitutional homozygosity at D5S346 or showing MSI in tumours were scored as non-informative.

### MSI analysis

MSI was assessed at five microsatellite loci (Bat-25, Bat-26, D2S123, D5S346, and D17S250) as described previously [18]. Matching normal and tumour DNA samples were compared, and tumours showing instability at one locus were scored as MSI-low (MSI-L), at two or more loci as MSI-high (MSI-H).

### DNA methylation assay

DNA methylation of the *MLH1* promoter was analysed using methylation specific multiplex ligation-dependent probe amplification (MS-MLPA). SALSA MS-MLPA Kit ME011-A1 (MRC-Holland, Amsterdam, The Netherlands) with 6 probes in the *MLH1* gene was used according to the manufacturer instructions. PCR products were analysed using an ABI 3130 Genetic Analyser. Data analysis was performed with the Genemapper and Coffalyser software (Applied Biosystems and MRC-Holland, respectively). The relative peak area of the signal from a specific probe was calculated by dividing the peak area by the combined areas of peaks of the control probes and multiplying the value by 100. The relative peak areas of probes from the Hha I digested sample were compared with those from the corresponding undigested sample, giving the percentage ratio of methylation at CpG sites. The cut-off value for aberrant methylation was set to 25% or higher.

### Immunohistochemistry (IHC)

A portion of each tissue sample was formalin fixed, embedded in paraffin and processed using standard histopathologic procedures. Representative blocks containing enough of tumour and normal tissue were cut to 4 µm sections, deparaffinised and rehydrated. Target Retrieval Solution, High pH (DakoCytomation, Glostrup, Denmark) was used for epitope retrieval at 96°C for 30 min. The sections were incubated overnight at 4°C with primary monoclonal mouse anti-human *MLH1* and *MSH2* antibodies (clones G168-15 and G219-1129, BD Biosciences, NJ, USA) diluted 1∶100. The *MLH1* antibody complexes were visualized using the streptavidin-biotin detection kit LSAB+, Dako REAL Detection Systems, HRP/DAB+, Rabbit/Mouse (DakoCytomation) and 3,3′-diaminobenzidin tetrahydrochlorid (DAB, Fluka Chemie, Buchs, Switzerland). The *MSH2* antibody complexes were localised using N-Histofine Simple Stain MAX PO (MULTI) (Nichirei Biosciences, Tokyo, Japan) and DAB. All sections were stained with hematoxylin, dehydrated and mounted. Nuclear staining only was considered for both antibodies, and the normal tissue in the same section was used as an internal positive control. Only cases with complete negativity of all tumour cells and positivity of the internal control were interpreted as negative and suspicious of *MLH1* or *MSH2* gene dysfunction. This IHC analysis was performed in 39 tumours.

### Statistical analyses

Statistical analyses were carried out using GraphPad InStat 3.10 (GraphPad Software, La Jolla, CA, USA) and IBM SPSS Statistics version 18 (IBM Corporation, New York, USA). Fisher's exact, Chi-square or Exhaustive CHAID tests were used where appropriate. All P values were two-tailed, and P values less than 0.05 were considered statistically significant. Mutations of the genes tested and their combinations, LOH, MSI and *MLH1* promoter methylation were correlated with age at tumour onset, gender, location of the tumour, and its histopathological characteristics.

## Results

### Distribution of genetic defects in tumours

Tumours were scored as *APC* mutated if they carried at least one clearly deleterious *APC* mutation (mutations leading to premature termination and missense mutations found in CRC but not in the corresponding mucosa, absent from dbSNP and predicted to be pathogenic - a total of 103 mutations). Overall, 71 tumours (68.9%) had the *APC* gene mutated (Table 1): 45 CRCs had 1 deleterious mutation, 23 had 2 mutations, 2 had 3 mutations and 1 had 6 mutations. Seven additional tumours (6.8%) showed no mutation but had LOH at the *APC* locus. Twenty tumours had neither a mutation nor LOH. The remaining 5 non-mutated tumours were not informative. Three missense *APC* variants (R382S, N813S and A1366V) were absent from mucosa and dbSNP but were predicted to be benign, totalling the number of somatic *APC* variants observed to 106.

**Table 1:** Numbers of tumours with genetic defects studied.

(n)	*APC* mutated	*KRAS* mutated	*TP53* mutated	*BRAF* mutated	*CTNNB1* mutated	with *MLH1* methylation	with MSI
	(%)	(%)	(%)	(%)	(%)	(%)	(%)
all CRC (103)	71	32	28	9	2	13	25
	(68.9)	(31.1)	(27.2)	(8.7)	(1.9)	(12.6)	(24.3)
Sex of the patient							
CRC in females (51)	36	14	15	4	1	7	11
	(70.6)	(27.5)	(29.4)	(7.8)	(2)	(13.7)	(21.6)
CRC in males (52)	35	18	13	5	1	6	14
	(67.3)	(34.6)	(25)	(9.6)	(1.9)	(11.5)	(26.9)
Tumour location							
proximal CRC (43)	26	10	7*	7*	0	13**	21**
	(60.5)	(23.3)	(16.3)	(14.9)	(0)	(30.2)	(48.8)
distal CRC (60)	45	22	21*	2*	2	0**	4**
	(75)	(36.7)	(35)	(3.3)	(3.3)	(0)	(6.7)
Tumour stage							
I (18)	14	5	7	1	0	1	4
	(77.8)	(27.8)	(38.9)	(5.6)	(0)	(5.6)	(22.2)
II (38)	24	12	2	5	0	9	14
	(63.2)	(31.6)	(5.3)	(13.2)	(0)	(23.7)	(36.8)
III (32)	23	8	12	2	2	2	4
	(71.9)	(25)	(37.5)	(6.3)	(6.3)	(6.3)	(12.5)
IV (12)	7	4	5	1	0	1	2
	(58.3)	(33.3)	(41.7)	(8.3)	(0)	(8.3)	(16.7)
n.a. (3)	3	3	2	0	0	0	1
Lymphatic invasion							
0 (56)	38	17	9*	6	0	10	18
	(67.9)	(30.4)	(16.1)	(10.7)	(0)	(17.9)	(32.1)
I+II (43)	29	11	17*	3	2	3	6
	(67.4)	(25.6)	(39.5)	(7)	(4.7)	(7)	(14)
n.a. (4)	4	4	2	0	0	0	1

n.a., information not available; * significant (P<0.05); ** significant (P<0.0001, Fisher's exact).

LOH at the *APC* locus was found in 35 of 90 informative tumours (38.9%). Of 63 informative tumours with the *APC* gene mutated, 28 had LOH at the *APC* locus (44.4%). Of 43 tumours with only one deleterious *APC* mutation, 27 had LOH (62.8%), while of 20 tumours with 2 and more mutations, only 1 had LOH (5%, P<0.0001). Mutant allele specific amplification was possible in 12 of 26 tumours with 2 and more *APC* mutations to show their phase. Fourteen other samples could not be assessed due to a large distance between the mutations (≥10 kb, 10 tumours; ≥5 kb, 4 tumours). In all 12 tumours tested the mutations were in trans configuration. Out of the 27 tumours with LOH and one *APC* mutation, 15 had LOH of the wildtype allele based on the relative signal intensity of the mutated and wildtype alleles, 6 had LOH of the mutant allele and 6 cases could not be unequivocally resolved. However, any admixture of normal mucosa can partly mask the LOH of the wildtype allele.

**Figure 1:**
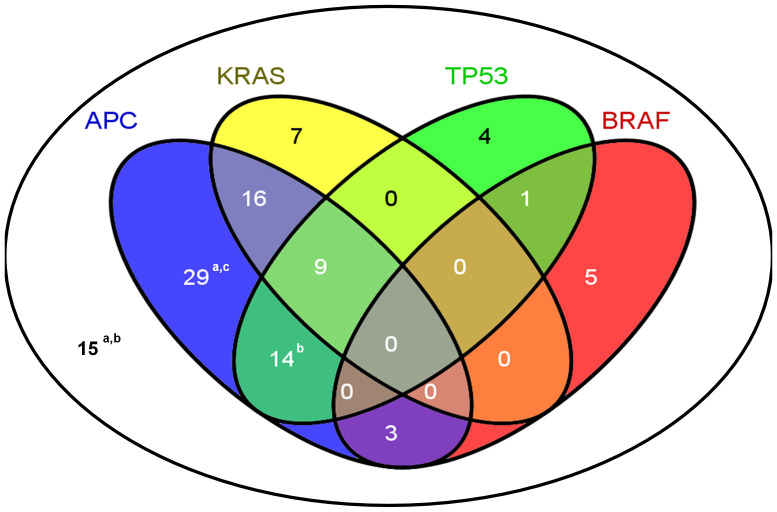
Distribution of mutations in selected genes. Mutational events in the *APC*, *TP53*, *KRAS* and *BRAF* genes dispersed in 103 tumours studied. Nineteen tumours carried no mutations in these genes. ^a^ this group includes 1 tumour with a *CTNNB1* mutation; ^b^ this group includes 1 tumour with a germline *MUTYH* mutation; ^c^ this group includes 1 tumour with 8 point substitutions in *APC*.

*TP53* mutations were considered deleterious if they were absent from mucosa and dbSNP, and were not listed as polymorphisms in UMD. Tumours with any variation in codons 12, 13 or 61 of *KRAS* and in exon 15 of *BRAF* were classified as mutated. In contrast to *APC*, no tumours contained more than one mutation in any of these 3 genes. Simultaneous occurrence of mutations in the *APC*, *TP53*, *KRAS* and *BRAF* genes is shown in Figure 1. The most common combination of mutated genes was *APC* and *KRAS* (35.6%), *APC* and *TP53* (31.1%), whereas no tumours had mutations in *TP53* and *KRAS* only. *BRAF* mutations were mutually exclusive with *KRAS* mutations, and four *BRAF* mutations were found in tumours with MSI. Fifteen tumours lacked mutations in any of these 4 genes; however, 3 of them had LOH in *APC*. Of the remaining 12 tumours, 2 were not informative for LOH and 10 were lacking any detectable defect in these 4 genes.

Only 2 tumours had mutations in the *CTNNB1* gene. One of them was the tumour with 6 deleterious *APC* mutations mentioned above. No other mutations or LOH were found in the two *CTNNB1* mutated tumours. Two other CRCs carried heterozygous *MUTYH* mutations which were also present in the mucosa, thus excluding the presence of MAP patients in our cohort.

MSI was found in 25 tumours (24.3%), out of which MSI-H in 21 cases (20.4%) and MSI-L in 4 cases (3.9%). Twelve tumours with MSI (all MSI-H) had the *MLH1* promoter methylated. None of the patients with a MSI tumour had a relative with a HNPCC-related cancer. Thirteen MSI tumours without *MLH1* methylation displayed more severe mutation profiles (11 had an *APC* defect, 4 *KRAS*, 2 *CTNNB1* and 3 *TP53* mutations, and only 1 showed no other genetic defect than MSI). This contrasted to 12 tumours with MSI and *MLH1* methylation, of which 7 had no other genetic defect or a *BRAF* mutation (P = 0.0099). Six of 9 *BRAF* mutated tumours showed MSI, and all of those had the *MLH1* promoter methylated and were proximally located.

### Mutation spectra

**Figure 2:**
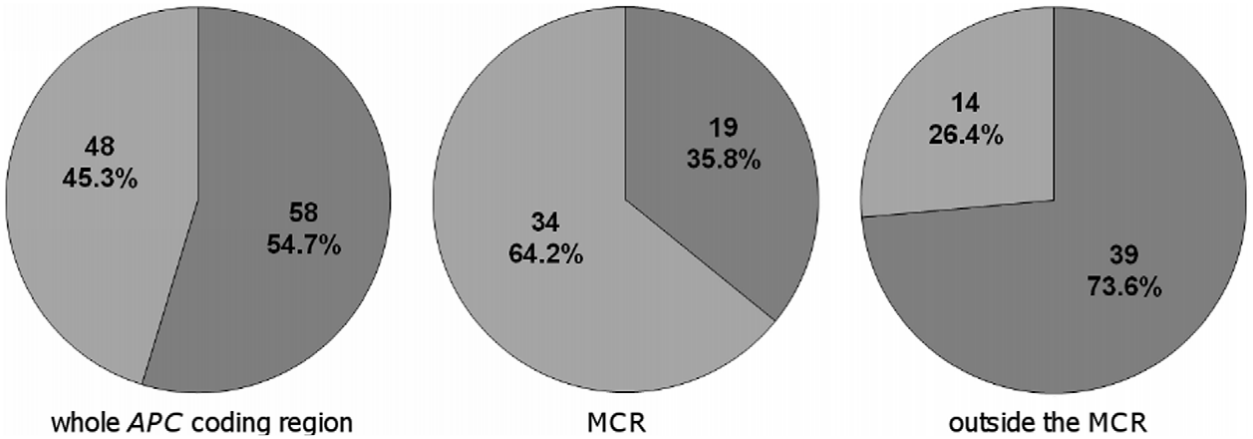
Comparison of the type of mutations in the *APC* gene. Light grey, frameshift mutations; dark grey, point substitutions. Frameshift mutations were more frequent in MCR while point substitutions, especially C>T resulting in Arg>STOP, were more common outside the MCR.

Considering all 106 variants found in *APC*, point substitutions (46 nonsense, 9 missense and 3 splice, 54.7%) were slightly more frequent than frameshift (FS) mutations (29 deletions, 18 insertions/duplications and 1 indel, 45.3%, Figure 2). However, in the mutation cluster region (MCR) [7], FS mutations occurred more often than point substitutions (34 FS (64.2%) and 19 point substitutions (35.8%) out of the total of 53 mutations in MCR, P = 0.0003, Figure 2). Of the total of 31 *APC* mutations in MSI tumours, 15 were FS (48.4%) and 16 point substitutions (51.6%). This was similar to tumours without MSI, where out of the total of 72 mutations 33 were FS (45.8%) and 39 point substitutions (54.2%, P = 0.8326).

**Figure 3:**
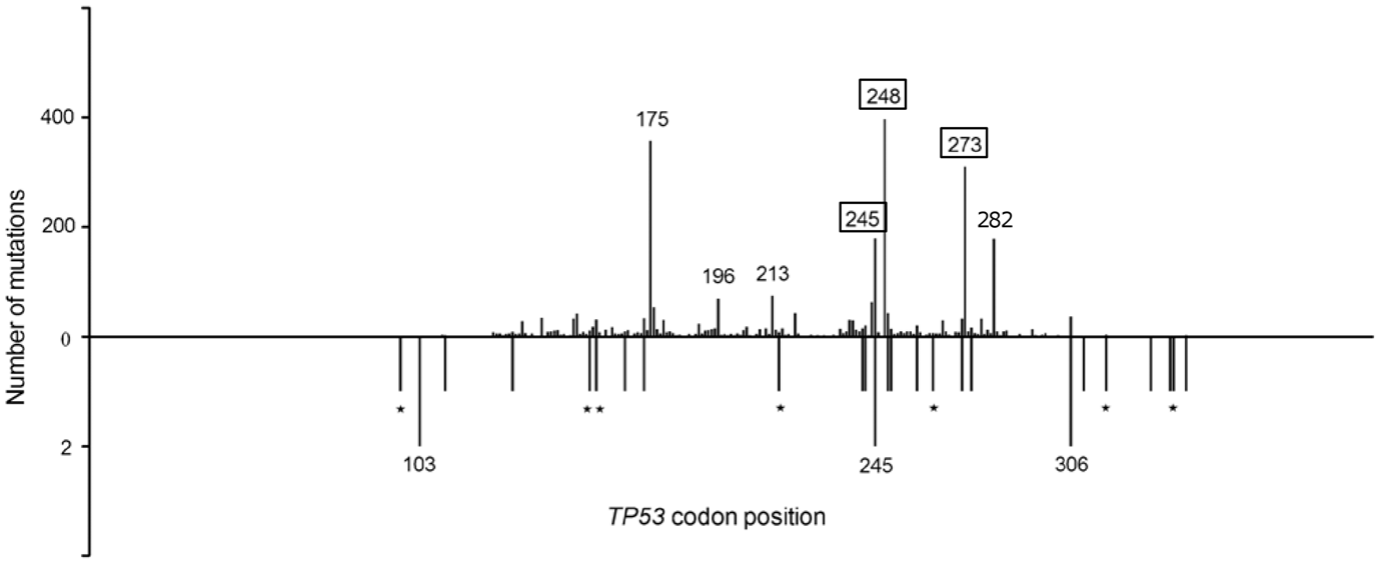
Comparison of the distribution of mutations along the *TP53* coding region. *TP53* mutations in CRCs from the UMD database (top) and in our sample (bottom) are depicted. The length of the bars reflects the number of mutations. Seven hot spot positions (representing 10 most common substitutions in UMD) are indicated by codon numbers. Numbers in frames indicates two different frequent substitutions at the same position. In our sample, two different point substitutions were observed in codon 245, and only one of them belonged to the 10 hot spot variants. Asterisks indicate FS mutations in our cohort.

**Figure 4:**
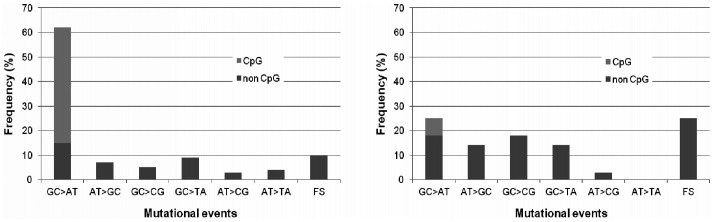
Mutational events in the *TP53* gene. Mutations found in CRCs listed in the UMD database (left) and observed in our CRCs (right). The CRCs of Czech patients had more frameshift mutations and transversions, while transitions, especially at CpG sites, were less frequent.

Of 28 *TP53* mutations observed, 15 (53.6%) were missense and 13 (46.4%) were FS and nonsense, which was significantly different from CRC mutations listed in UMD, where missense mutations were highly predominant (81% missense, 19% FS and nonsense, P = 0.0005, X^2^ test). *TP53* mutations were located in exons 4-10, with 64% of them in exons 5-8. Interestingly, only 1 *TP53* mutation (4%) belonged to the 10 most common CRC mutations listed in UMD, where out of 3584 mutations 1566 (43.7%) were in these hotspots (P = 0.004, X^2^ test, Figure 3). Compared to UMD, we could observe less G∶C>A:T but more G∶C>C∶G, G∶C>T∶A and FS events (Figure 4).

The majority of *KRAS* mutations, 23 (71.9%), were found in codon 12. Codons 13 and 61 were involved in 5 (15.6%) and 4 (12.5%) tumours, respectively. The most common substitution was G>A (16, 50%), G>T (11, 34.4%) and A>C (3, 9.4%), while G>C and A>T substitutions were present once each (3.1%).

No difference in tumour spectra was observed between males and females.

### Correlation of molecular findings with clinical and IHC data

**Figure 5:**
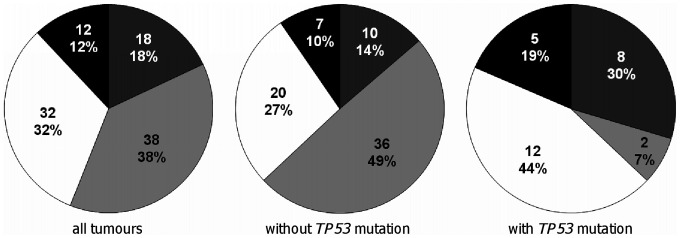
Comparison of the stage of tumours without and with *TP53* mutations. White, stage I; light grey, stage II; dark grey, stage III; black, stage IV. Segments show the number and percentage of tumours (3 tumours lacked information). Tumours with *TP53* mutations may have a tendency to skip stage II or progress through it very quickly compared to tumours without *TP53* mutations (P = 0.002).

*APC* and *KRAS* mutations did not show any significant correlation with tumour location, stage, grade and lymph node involvement, age at onset or sex of the patient. *BRAF* mutations were correlated with proximal tumour location: 7 of 36 proximal (19.4%) and 2 of 58 distal (3.5%) tumours were mutated (P = 0.0323). *TP53* mutations were more frequent in distal tumours: 21 of 60 distal tumours (35.0%) were mutated compared to only 7 of 43 proximal tumours (16.3%, P = 0.0440), and in invading tumours: 17 of 43 invading tumours (39.5%) were mutated compared to only 9 of 56 non-invading tumours (16.1%, P = 0.0114, 4 tumours lacked information). Exhaustive CHAID test revealed that tumours with *TP53* mutations had a tendency to skip stage II or progress through it very quickly to stage III (P = 0.002, Figure 5).

The number of *CTNNB1* mutated tumours was too low for any correlation. Both were in distal colon and showed lymphatic invasion. Concerning *MUTYH,* one mutation carrier was an 83 year old woman with stage II, grade 2, proximally localized CRC without lymphatic invasion and MSI but with 2 FS *APC* mutations and one splice *TP53* mutation. The other *MUTYH* mutation carrier was a 58 year old man with proximally localized stage III, grade 3 CRC, with no lymphatic invasion, showing MSI-H and *MLH1* methylation but no other genetic defects.

Both MSI and *MLH1* promoter methylation were significantly more frequent in proximal tumours (MSI in 21 of 43 proximal tumours but only in 4 of 60 distal tumours, P = <0.0001; *MLH1* methylation in 13 of 30 proximal tumours but in none of 60 distal tumours, P = <0.0001, Table 1). The mean age at tumour onset was similar in patients with and without MSI (62.7 and 64.7 years, respectively, P = 0.4723). However, the mean age at onset in 13 patients with MSI without *MLH1* methylation was lower (58.2 years) compared to that in 12 patients with MSI and *MLH1* methylation (67.6 years, P = 0.0894). The tumour stage was comparable in both groups. The tumour grade was predominantly I+II in the first group and III in the second (P = 0.0820).

## Discussion

We analysed mutation profiles in sporadic CRCs of Czech patients, in whom CRC incidence and mortality is one of the highest in Europe and is still increasing [1], [19].

*APC* mutations are the key player in CRC tumorigenesis. However, mutation analysis of the *APC* gene is time-consuming and expensive, and is often limited to MCR which covers about 10% of the APC coding region [7]. In several studies where the entire gene was sequenced the frequency of mutations in CRCs was 60% [15], [20], similar to our results (68.9%). If we restricted our analysis to MCR only, we would miss 59 mutations (55.6%) including 33 mutations in 26 tumours (25.2%) which would be classified as non-mutated. Some reports indicated an interdependence of two hits in *APC* both in sporadic and in FAP associated CRCs: *APC* mutations in the MCR were predominantly associated with LOH while mutations outside the MCR with another mutation [21], [22]. In our cohort, mutations in the MCR and outside the MCR were equally associated with LOH, and many mutations outside the MCR were coupled with at least one mutation in the MCR. Point substitutions occurred more often outside the MCR compared to FS mutations (Figure 2), and commonly included C>T transitions at CpG sites mainly changing arginine codons to STOP as reported previously [15], [20]. The frequency of *APC* defects rose to 75.7% if LOH at the *APC* locus was included (7 of our CRCs had LOH only). LOH at the *APC* locus was reported in 30-40% of CRCs [22], similarly to our results (35%). Significantly increased LOH in our tumours with just 1 somatic *APC* mutation, the trans position of 2 pathogenic *APC* mutations confirmed in tumours where the mutation phase was tested, and preferential loss of the wildtype allele in tumours with LOH and an *APC* mutation support the two-hit model.

CRCs with intact *APC* may carry mutations in *CTNNB1*, a critical downstream gene of the WNT signalling pathway [23], although these are rather rare in sporadic CRCs [9], [10]. *CTNNB1* mutations may be more frequent in MSI-H tumours [9]. Both our tumours with *CTNNB1* mutations were MSI-H. One carried p.S45F, a likely activating mutation located in one of the hotspots and supposed to deregulate the WNT signalling instead of *APC*. Indeed, this tumour carried no *APC* mutation. The other CRC had the p.A20V mutation, which is not located in any of the critical sites, does not change amino acid polarity and may not be disease-causing. This tumour had several inactivating *APC* mutations.

Germline *MUTYH* mutations have lower incidence in the Czech Republic [5], [6]. The frequency of biallelic carriers of 2 most prevalent Caucasian mutations, p.Y179C and p.G396D, among *APC* negative patients with polyposis was 2-40%, and the frequency of carriers of monoallelic *MUTYH* mutations among CRC patients was 0.9-4.2% [5]. Cases of biallelic *MUTYH* carriers with sporadic CRC without polyps are rare [24], and our study included primarily individuals without polyps. Of the monoallelic *MUTYH* mutations observed in our patients, the p.R182H mutation is pathogenic [25], while p.Q479L is of an unknown effect. An elevated risk of CRC was proposed for carriers of monoallelic *MUTYH* mutations [26], but two large studies did not confirm this conviction [27], [28]. A retrospective analysis of pathological reports revealed 3 diminutive tubulovillous adenomas with moderate dysplasia in the carrier of the first mutation. However, neither of the 2 CRCs in our patients had the characteristic mutation profile [4], and therefore they were not likely caused by the germline *MUTYH* mutations.

In addition to "hidden" MAP patients the mutation analysis of the whole *APC* gene could also reveal other specific mutation profiles typical for Czech patients. We identified a remarkable patient with 8 somatic variants in the *APC* gene. Two of these point variants were nonsense, two splice, three missense (one of them predicted to be benign) and one silent, and, interestingly, the tumour was MSI-H. The patient was analysed in detail and the results will be published separately (manuscript in preparation).

MSI can be detected in up to 15% of sporadic CRCs and in almost all HNPCC-associated CRCs. While MSI in HNPCC tumours is caused by germline mutations in mismatch repair (MMR) genes, MSI in sporadic tumours is often associated with *MLH1* promoter methylation and accompanied by somatic *BRAF* mutations [14]. These changes were rarely if ever seen in HNPCC tumours, and may be mutually exclusive with *KRAS* mutations [29]. In our sample 24% of tumours showed MSI. This higher incidence can be explained by the exclusion of rectal tumours with neoadjuvant therapy that biased the distribution towards proximal tumours where the MSI frequency is higher [30]. Similarly also the exclusion of tumours with no available matching mucosa might have biased the sample against aggressive and rectal cancers. In our cohort the frequency of *MLH1* promoter methylation decreased with the distance of the tumour from caecum, and was completely absent in tumours of the distal colon. Rare *MLH1* promoter methylation in rectal cancers was described in one study, but it was accompanied by high rate of MMR protein deficiency, possibly due to the inclusion of Lynch-associated tumours [31]. The rectal MSI-H tumours had worse prognosis compared to those without MSI, which could be caused by pre-operative irradiation or chemotherapy which had no effect or might even be harmful for MSI-H cancer patients [31]. In another study, distal MSI CRCs had lower incidence of *MLH1* methylation and worse prognosis compared to proximal MSI CRCs [32]. Our data suggest that proximal CRCs without *MLH1* methylation could have similar clinicopathological and molecular features as distal CRCs. Although we did not perform IHC analysis of all MMR proteins in all MSI tumours and therefore cannot exclude germline mutations in these genes, we suppose that tumours without *MLH1* methylation represent a different subgroup. The reason for the uneven localization of the MSI tumours, differential *MLH1* promoter methylation and earlier onset of MSI CRCs without *MLH1* methylation is unknown and can be caused by dietary habits, different environment (e.g. varying pH) in different parts of the colon, different genes involved or the combination of all of the above.

Our data confirmed the notion that *BRAF* mutations are frequently found in sporadic MSI tumours [29], and support the previous observation of *BRAF* mutations in about 5% of CRCs without MSI [29], [33]. In accordance with other reports [34], [35], we have found the inverse association between *BRAF* and *APC* mutations. Six of 9 tumours with a *BRAF* mutation had no somatic *APC* mutation. None of these 9 tumours had a *KRAS* mutation either, which is in accord with others [33], [34]. We did not observe any differences in clinicopathological features of these tumours. MMR deficiency, irrespective of its genetic or epigenetic origin, leads to the mutator phenotype, and FS *APC* mutations, predominantly in mononucleotide tracks, are more frequent in MSI tumours [36]. The mutational spectrum of our MSI tumours was not different from that in tumours without MSI, but FS mutations were more frequent in proximal MSI tumours without *MLH1* promoter methylation.

The increased incidence of CRC in the Czech Republic can be partly explained by the joint effect of elevated smoking prevalence and obesity [19], [37]. Mutation signatures in *TP53* can reflect DNA damage induced by specific carcinogens, ethnicity or lifestyle habits [12]. For example, exposure to ultraviolet light is correlated with *TP53* transitions at dipyrimidine sites (CC>TT) [12]; aflatoxin B_1_ exposure with G∶C>T∶A transversions in codon 249 in hepatocellular carcinoma; and exposure to cigarette smoke with G∶C>T∶A transversions in lung carcinoma [12]. *TP53* mutations observed in our sample included very few hotspot codons, and their pattern and distribution was distinct from that of CRC mutations listed in UMD (Figures 3, 4). Czech CRC patients have less G∶C>A∶T transitions and more FS, G∶C>C∶G and G∶C>T∶A events, although no predominant mutational event or specific hot spot can be observed. These mutations are caused by polycyclic aromatic hydrocarbons (PAHs) [38], [39]. One of the main sources of PAHs except tobacco smoke and environmental pollutants is high-fat diet rich for smoked red meat [40]. PAHs are formed on the surface of meat at high temperatures [41]. Home production of smoked food and high consumption of red meat products is characteristic of Czech households, especially in rural areas [42]. It remains to be verified on a larger set of CRCs if these dietary habits are the cause of the *TP53* mutation signature observed. Another explanation could involve population differences in the frequency of functional polymorphisms in DNA repair genes, which could modify the risk of CRC [43]. Further research is needed to address this scenario.

Frequency and spectrum of *KRAS* and *APC* mutations did not differ compared to most other reports. This could be partly explained by the nature of mutations in these genes and their ability to give the cell a growth advantage leading to positive clonal selection. Only *KRAS* mutations in codons 12, 13, and 61 and nonsense *APC* mutations are considered to give such advantage, and therefore the investigation of mutational spectra of these genes is of limited use [44].

No tumours had concurrent *TP53* and *KRAS* mutations in context of non-mutated *APC* (Figure 1). Similar findings were noted in 2 other studies [15], [45]. Concurrent *TP53* and *KRAS* mutations could be disadvantageous for tumour progression and may arise only on the *APC* mutation background.

Nowadays two main independent molecular pathways of colorectal tumorigenesis have been proposed: the conventional adenoma-carcinoma pathway characterised with the initial inactivation of the *APC* gene, accumulation of mutations in other genes and chromosomal instability [46]; and the serrated pathway with microsatellite instability, a relatively high frequency of *BRAF* mutations and increased level of DNA methylation [47]. Although most of CRCs could be clearly classified into one of these pathways, they overlap and the mutational profile of a CRC may show evidence of both. Thus, the classification of many tumours remains ambiguous, e.g. of MSI tumours without *MLH1* promoter methylation but with severe mutational profiles and earlier onset of the disease compared to Lynch associated tumours, as reported here and elsewhere [31], [32]. Although we cannot exclude the possibility that among 13 MSI tumours from our cohort without *MLH1* promoter hypermethylation there may be a hidden Lynch associated tumour, the selection criteria and the relatively low percentage of true Lynch tumours among unselected CRCs [48] stand against it.

There is an increasing effort to assess individual specific molecular alterations for personalized diagnosis, prognosis and/or treatment. As can be seen from our results, tumours with mutations in multiple genes often had better staging or grading compared to tumours with no or only very few genetic defects. This implies that focusing on a single gene or defect or interpretation of the findings using too simple rules may be misleading. Systematic sequencing of cancer genomes reveals the diversity of cancer as to the number and pattern of mutations arising probably due to DNA repair defects, mutagenic exposure and cellular metabolism [49]. It has been shown that a single CRC can harbour up to 76 point mutations and 9 copy number changes, and that rather whole pathways than individual genes govern the process of carcinogenesis [50], [51]. High-throughput methods like next-generation sequencing or copy number variation arrays can therefore be more helpful in managing cancer patients.

In summary, the molecular genetic analysis of CRCs in Czech patients confirmed the data from other studies but also yielded potentially novel findings. First, MSI tumours with unmethylated *MLH1* promoter have earlier onset and more severe mutational phenotype. Second, the Czech pattern and distribution of *TP53* mutations differ significantly from published data. Third, mutational analysis of the whole coding region of the *APC* gene significantly increases the yield of the analysis, but did not pinpoint any MAP patient, confirming that germline *MUTYH* mutations are rare in the Czech population.
