# Correction: Molecular Genetic Analysis of 103 Sporadic Colorectal Tumours in Czech Patients

**DOI:** 10.1371/journal.pone.0351709

**Published:** 2026-06-16

**Authors:** The PLOS One Editors

## Notice of Republication

This article [1] was republished on February 12, 2026, to address an issue identified post-publication. An updated version of S1 Table is provided with this notice. Please download this article again to view the correct version.

The following sentence should be added as the final sentence of the Patients subsection of the Materials and Methods section: The characteristics of the patients and clinical and histopathological features of their tumours are shown in S1 Table.

The following sentence should be added as the first sentence of the Results section: Details of mutation profiles, LOH, MSI, DNA methylation and IHC of individual tumours are shown in S1 Table.

Additionally, references to S1 Table should be added to the below sentences as follows:

- The final sentence of the Immunohistochemistry (IHC) subsection of the Materials and Methods section: This IHC analysis was performed in 39 tumours (see S1 Table).
- The final sentence of the third paragraph of the Mutation spectra subsection of the Results section: No difference in tumour spectra was observed between males and females (S1 Table).
- The first sentence of the first paragraph of the Correlation of molecular findings with clinical and HIC data subsection of the Results section: *APC* and *KRAS* mutations did not show any significant correlation with tumour location, stage, grade and lymph node involvement, age at onset or sex of the patient (S1 Table).
- The seventh sentence of the sixth paragraph of the Discussion section: In our cohort the frequency of *MLH1* promoter methylation decreased with the distance of the tumour from caecum, and was completely absent in tumours of the distal colon (S1 Table).

## Supporting information

S1 Table The characteristics and clinical features of the patients and mutation profiles, LOH, MSI, MLH1 methylation, IHC and histopathology of their tumours. (XLS)
